# Screening and functional analysis of differentially expressed genes in EBV-transformed lymphoblasts

**DOI:** 10.1186/1743-422X-9-77

**Published:** 2012-03-30

**Authors:** Yongming Dai, Yunlian Tang, Fei He, Yang Zhang, Ailan Cheng, Runliang Gan, Yimou Wu

**Affiliations:** 1 Cancer Research Institute, University of South China, Hunan, 421001, People’s Republic of China; 2 Clinical Laboratory, The Third People Hospital of Kunshan City, Jiangsu, 215300, People’s Republic of China; 3 Shanghai Center for Bioinformation Technology (SCBIT), Shanghai, 200235, People’s Republic of China; 4 Pathogenic Biology Institute, University of South China, Hunan, 421001, People’s Republic of China; 5 Cancer Research Institute, University of South China, School of Medicine, Chang Sheng Xi Avenue 28, Hengyang City, Hunan, 421001, People’s Republic of China; 6 Pathogenic Biology Institute, University of South China, Hengyang, 421001, China

**Keywords:** Epstein-Barr virus (EBV), Lymphocyte transformation, Lymphoblastoid cell line (LCL), Gene expression, Gene chip

## Abstract

**Background:**

Epstain-Barr virus (EBV) can transform human B lymphocytes making them immortalized and inducing tumorigenic ability *in vitro*, but the molecular mechanisms remain unclear. The aim of the present study is to detect and analyze differentially expressed genes in two types of host cells, normal human lymphocytes and coupled EBV-transformed lymphoblasts *in vitro* using gene chips, and to screen the key regulatory genes of lymphocyte transformation induced by EB virus.

**Methods:**

Fresh peripheral blood samples from seven healthy donors were collected. EBV was used to transform lymphocytes *in vitro*. Total RNA was extracted from 7 cases of the normal lymphocytes and transformed lymphoblasts respectively, marked with dihydroxyfluorane after reverse transcription, then hybridized with 4 × 44 K Agilent human whole genome microarray. LIMMA, String, Cytoscape and other softwares were used to screen and analyze differentially expressed genes. Real-time PCR was applied to verify the result of gene expression microarrays.

**Results:**

There were 1745 differentially expressed genes that had been screened, including 917 up-regulated genes and 828 down-regulated genes. According to the results of Generank, String and Cytoscape analyses, 38 genes may be key controlled genes related to EBV-transformed lymphocytes, including 22 up-regulated genes(PLK1, E2F1, AURKB, CDK2, PLCG2, CD80, PIK3R3, CDC20, CDC6, AURKA, CENPA, BUB1B, NUP37, MAD2L1, BIRC5, CDC25A, CCNB1, RPA3, HJURP, KIF2C, CDK1, CDCA8) and 16 down-regulated genes(FYN, CD3D, CD4, CD3G, ZAP70, FOS, HCK, CD247, PRKCQ, ITK, LCP2, CXCL1, CD8A, ITGB5, VAV3, CXCR4), which primarily control biological processes such as cell cycle, mitosis, cytokine-cytokine pathway, immunity response and so on.

**Conclusions:**

Human lymphocyte transformation induced by EB virus is a complicated process, involving multiple-genes and –pathways in virus-host interactions. Global gene expression profile analysis showed that EBV may transform human B lymphocytes by promoting cell cycle and mitosis, inhibiting cell apoptosis, hindering host immune function and secretion of cytokines.

## Background

Epstain-Barr virus (EBV) is closely associated with many kinds of human tumors, including nasopharyngeal carcinoma, Burkitt lymphoma, Hodgkin's lymphoma, post-transplant lymphoproliferative disorders (PTLD) and others [1-3]. EBV can localize to B cells during latent infections, since cells in this phase only express EBV latent proteins with weak immunogenicity, thereby inhibiting the body’s ability to recognize and eliminate EBV-infected cells. Under certain conditions and the actions of some inducing factors, the latent infecting EBV is reactivated, which can induce abnormal proliferation or transformation of B cells, and might induce the genesis of EBV associated tumors in some cases [1].

EBV can transform human B lymphocytes making them immortalized and inducing tumorigenic ability *in vitro*[4], but the molecular mechanisms remain unclear. In this study, normal peripheral blood lymphocytes (PBLs) and coupled EBV transformed lymphoblastoid cell lines (LCLs) were used as experimental models to screen and compare the differentially expressed genes of the two types of host cells *in vitro*, using human whole genome microarrays, which possess different characteristics and the same genetic background, and to analyze the biological functions and interactions. Molecular regulatory networks for EBV-induced human lymphocyte transformation were constructed to reveal the key genes involved in the malignant transformation of EBV-infected lymphocytes.

## Materials and methods

### Preparation of EBV suspension

B_95-8_ cells with well-defined morphologies were selected, and the cell concentration was adjusted to 10^6-7^/ml after the last change of culture medium. Cells were then incubated at 37°C and 5% CO_2_ incubator for 7 ~ 10 d. The cultured solution was collected on the 10^th^ day, and centrifuged at 4000 r/min and 4°C for 30 min. The supernatant was filtered through 0.45 μm microporous membranes, subpackaged and sealed in freezing tubes and stored at −80°C.

### Establishment of LCLs

Fresh peripheral blood samples from seven healthy adult donors were collected. Detection of EBV-VCA-IgG kit (ELISA) for the serum and PCR amplification of EBV-LMP1 in the whole blood DNA were used to verify EBV infection status of the seven blood donors. All of the seven donors were EBV-positive.

Normal PBLs were separated from fresh peripheral blood samples using human lymphocyte separation medium (Cat #LTS1077, Tian Jin Hao Yang Biological Manufacture Co., Ltd.) and washed twice with RPMI 1640. A portion of the obtained PBLs were lysed with Trizol (Invitrogen, USA) and preserved at −80°C, whereas the remaining PBLs were used for EBV transformation according to the method described by David [5]. Briefly, 2 ~ 3 × 10^6^ normal PBLs were resuspended in RPMI 1640 which contained 2 ml cyclosporine A ([Sandoz], with a final concentration of 2 μg/ml) and 25% fetal bovine serum, and then inoculated to two wells of a 24-well plate. One ml of EBV suspension was added to each well. A week later, lymphoblastoid-like changes in cell morphology, volume enlarging, and cell aggregation were observed. One-half of the cultivating mediums were exchanged every 3 ~ 4 d. After four weeks, the cells were transferred to a 25 ml culture bottle for subculturing. Resuscitation activity of seven samples of transformed cells that were stored in liquid nitrogen for four weeks was well, and could be used for continuous subculturing. The successfully transformed LCLs were lysed with Trizol and stored at −80°C.

### Screening and analysis of differentially expressed genes

Total RNA was extracted from seven cases of the normal lymphocytes and transformed lymphoblasts, respectively, marked with dihydroxyfluorane after reverse transcription, then hybridized with Agilent whole genome microarray (Design ID. 14850). Agilent whole genome microarray and hybridization experiment were provided and completed by Kangchen Bio-tech Inc (Shanghai, China). Agilent whole genome microarray included 41000 genes. Sixty-mer oligonucleotide probes were manufactured using a computer-driven inkjet printing process with Sureprint technology. Probe designed was based on database resources including Genbank, Ensemble and others, and was screened and optimized following well-tested protocols. The microarrays were scanned using Agilent Microarray Scanner (G2505B), with Agilent Feature Extraction and Agilent Genepix GX software. LIMMA was used to screen differentially expressed genes. Cluster 3.0 and Tree View software were used for cluster analyses. GO and Pathway software were used for analysis of gene function. Generank was used for gene ranking, and String and Cytoscape software were used to draw genetic interaction network.

### Real-time PCR verification

Four genes of interest were randomly selected from differentially expressed genes screened in the above experiments. Real-time PCR was performed with GADPH as an internal control to verify the microarray results. Primer sequences (5'-3') were as follows: E2F1: upstream GAACTGGGCTGCCGAGGT, downstream AGGACGTTGGTGATGTCATAGAT; PLK1: upstream CAAGTGGGTGGACTATTCGGA, downstream CATTGTAGAGGATGAGGCGTGT; BIRC5: upstream GAGTCCCTGGCTCCTCTACTG, downstream TCACTGGGCCTGTCTAATTCAC; FYN: upstream GGAAGGAGATTGGTGGGAA, downstream CACGGATAGAAAGTGAATAGGC; GADPH: upstream GGGAAACTGTGGCGTGAT, downstream GAGTGGGTGTCGCTGTTGA. Both normal lymphocytes and transformed lymphoblasts were verified for seven samples, and each experiment was repeated three times.

## Results

### EBV-transformed lymphoblastes

**Figure 1:**
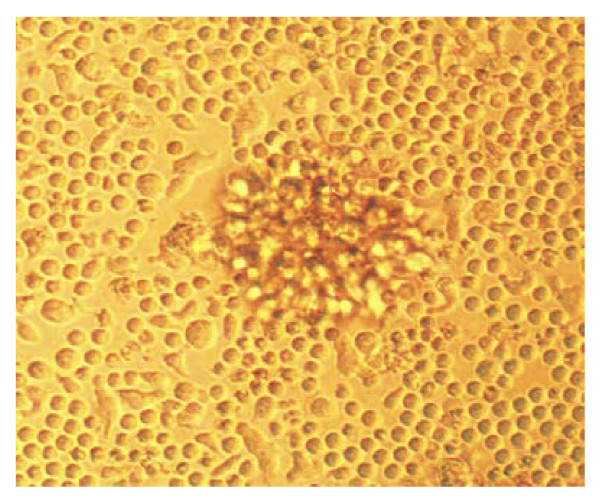
**EBV-transformed lymphoblasts*in vitro*(×200).** The volumes of successfully transformed lymphoblasts were significantly enlarged, rounded or ellipsed, where some had filiform antenna or pseudopodia. Cells aggregated into clusters and could be subcultured *in vitro* infinitely.

Morphological observation of successfully transformed LCLs by inverted microscopy showed that cells were rounded with obviously enlarged volumes, in which some cells showed filiform antenna or pseudopods, clustered and suspended growth. Occasionally tens or hundreds of cells aggregated into cluster [5], and could be subcultured *in vitro* infinitely (Figure 1). RT-PCR results of EBV-LMP1 of each cases of the LCLs derived from the seven donors was positive.

### Screening of differentially expressed genes

Microarray hybridization signal scanning showed that fluorescence signaling was strong and uniform, quality control was good, signal to noise ratio was low and the signal intensity reflected the RNA expression of the samples. All the raw data was standardized by robust quantile, then LIMMA software with false positive rate less than 0.0001 and two-fold changes in differential expression as a standard was used in screening genes. Results showed that there were 1,745 significantly differentially expressed genes in each of the seven samples, including 917 up-regulated genes and 828 down-regulated genes.

### Biological function analysis of differentially expressed genes

**Figure 2:**
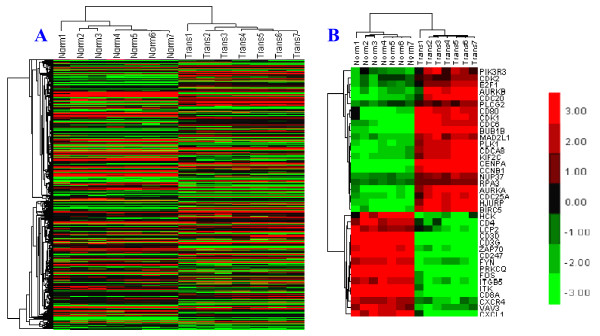
**Heatmap of differentially expressed genes. (A)** Heatmap of 1745 differentially expressed genes. Norms 1 ~ 7: normal lymphocytes 1 ~ 7, Trans 1 ~ 7: EBV-transformed lymphoblasts 1 ~ 7. Dendrograms showed that the differentially expressed genes ultimately clustered into two major branches, which indicated that the differentially expressed genes could distinguish normal PBLs and LCLs, and there existed significant difference between the expression patterns of two kinds of host cell genes. **(B)** Heatmap of 38 key controled genes. the 38 genes also clustered into two major branches, including 22 up-regulated genes and 16 down-regulated genes, and the expression of each genes can be notified. Up-regulated genes were labeled in red, down-regulated genes were labeled in green, genes showed no significant expression difference were labeled in black both in A and B.

Hierarchical cluster analysis of differentially expressed genes showed that the genes ultimately clustered into two major branches, which indicated that the differentially expressed genes were well distinguished between normal PBLs and LCLs, and there existed significant difference between the expression patterns of two kinds of host cell genes (Figure 2A).

**Figure 3:**
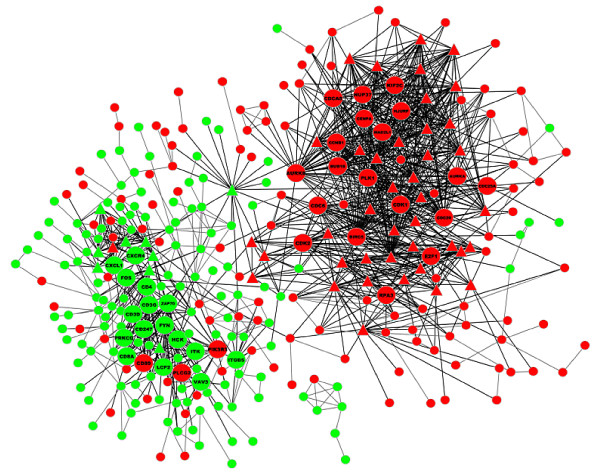
**Interaction networks maps of differentially expressed genes.** Up-regulated genes were labeled in red, down-regulated genes were labeled in green. ▵ labeled genes had ≥ 10 joint-edges, labeled nodes were the 38 genes that ranked the top 100 of up-regulated genes or the top 100 of down-regulated genes in the Generank rank. There were 88 nodes with ≥ 10 joint-edges in the map, including 1051 joint-edges, which accounted for 81% of the total.

Drawing and analysis of differentially expressed genes interaction networks. Differentially expressed genes were weight-sequenced according to the Generank algorithm (Table 1). Generank was based on the premise that nodes with more multiple of differentially expression and joint-edges have more important biological function [6,7]. Therefore, genes with the more advanced rankings were more biologically important in the process of EBV-transformation of lymphocytes. The 1,745 differentially expressed genes were mapped to the "experiment" and "database" sublibraries using String online software, and the Confidence Score was set at a high confidence level of 700. Results showed that there existed interactions among 350 nodes with a total of 1,292 joint-edges, and there were 88 nodes with ≥ 10 joint-edges, including 1051 joint-edges, accounting for 81% of the total, which suggested that the 88 genes may have important functions in EBV-transformed B lymphocytes (Figure 3). Among these 88 nodes, 38 were either ranked in the top 100 up-regulated genes or the top 100 down-regulated genes in Generank rank, which included 22 up-regulated genes and 16 down-regulated genes (Figure 2B). Annotation and analysis results of Genecards and Pathway showed that the up-regulated genes were mainly related to cell cycle, mitosis, DNA replication, and apoptosis regulation, whereas the down-regulated genes were mainly related to immunocyte activation, MHC-II/TCR/BCR expression and interaction, and cytokine interaction (Table 1).

**Table 1:** Functional annotation of 38 differentially expressed genes

**Notes**	**Generank**	**Fold Change**	**Edges**	**Gene Annotation**
**Up-regulated genes**				
PLK1	12.04	5.44	49	regulates M phase of cell cycle
E2F1	10.51	3.01	18	mediate cell proliferation and p53-dependent/indepent apoptosis
AURKB	10.07	5.13	39	mitosis checkpoint
CDK2	9.34	2.30	29	cell cycle checkpoint
PLCG2	8.93	2.18	10	crucial enzyme in transmembrane signaling
CD80	8.48	5.09	10	costimulatory signal for T cell activation
PIK3R3	7.39	2.94	15	responsible for a range of cell functions
CDC20	7.30	5.14	36	regulates multiple points of cell cycle
CDC6	6.90	5.75	26	initiates of DNA repliaction
AURKA	6.88	5.27	21	mitosis checkpoint
CENPA	6.87	6.87	28	mitosis checkpoint
BUB1B	6.81	6.55	34	mitosis checkpoint
NUP37	6.37	3.08	29	mitosis checkpoint
MAD2L1	6.32	4.08	16	spindle-assembly checkpoint
BIRC5	6.04	5.53	36	negative regulator of apoptosis
CDC25A	6.00	4.77	16	cell cycle checkpoint
CCNB1	5.81	5.81	18	cell cycle checkpoint
RPA3	5.64	2.42	17	required for DNA recombination, repair and replication
HJURP	5.62	5.62	13	fuctions in chromosome segregation
KIF2C	5.59	4.70	27	fuctions in chromosome segregation
CDK1	5.50	5.50	38	cell cycle checkpoint
CDCA8	5.45	5.27	26	mitosis checkpoint
**Down-regulated genes**				
FYN	−19.27	−4.92	34	involved in B/T cell activation
CD3D	−12.04	−10.50	19	involved in T/B-cell activation
CD4	−11.92	−4.97	20	MHC-II/T-cell receptor interacton
CD3G	−11.91	−9.81	19	involved in T-cell activation
ZAP70	−11.69	−6.20	21	involved in T-cell development and activation
FOS	−11.29	−8.32	14	involved in AP-1 and BCR/TCR signal pathway
HCK	−10.11	−5.28	17	contributes to neutrophil migration and degranulation
CD247	−9.16	−9.16	19	regulates TCR expression and signal transduction
PRKCQ	−9.10	−7.15	10	involved in T-cell activation
ITK	−8.93	−9.22	15	involved in T-cell proliferation and differentiation
LCP2	−8.53	−4.27	14	involved in T-cell antigen receptor mediated signaling
CXCL1	−8.19	−8.19	13	has chemotactic activity for neutrophils
CD8A	−8.08	−9.86	15	involved in T-cell mediated killing
ITGB5	−7.72	−5.16	11	receptor for fibronectin
VAV3	−7.60	−4.55	10	plays an important role in angiogenesis and FcϵRI signaling pathway
CXCR4	−7.20	−4.22	10	cytokine-cytokine receptor interaction

Note: Annotation and analysis results showed that the up-regulated genes were mainly related to cell cycle, mitosis, DNA replication, apoptosis regulation and etc., and the down-regulated genes were mainly related to T-cell (immunocytes) activation, MHC-II/TCR/BCR expression and interaction, cytokines interaction and etc.

### Results of real-time PCR verification

Real-time PCR results showed that trends in expression change for the randomly selected four genes were consistent with the microarray results. PLK1, E2F1, BIRC5 showed a high expression level in LCLs, and low expression in FYN (Table 2), verifying that the microarray results were accurate and reliable.

**Table 2:** Results of real-time PCR verification

**Gene**	**Cell**	**Number**	**Calc Conc**	***P-value***
E2F1	Norms	7	7.97 ± 0.15	1.55E-11
	Trans	7	88.49 ± 1.75	
PLK1	Norms	7	2.12 ± 0.16	6.65E-19
	Trans	7	15.97 ± 0.33	
BIRC5	Norms	7	2.01 ± 0.10	7.88E-10
	Trans	7	14.54 ± 0.58	
FYN	Norms	7	162.16 ± 2.92	2.83E-12
	Trans	7	6.35 ± 0.48	

Note: Norms--normal lymphocytes; Trans--transformed lymphoblasts. Calc Conc--calculation concentration.

## Discussion

The accumulation of high-throughput gene and protein expression profile data has greatly promoted the application of computer science and technology in functional gene studies. However, complex biological functions are not simply controlled by a single gene, as certain biological processes are realized by the cooperation of a series of genes. In this study, normal peripheral blood lymphocytes (PBLs) and coupled EBV-transformed lymphoblastoid cell lines (LCLs) were analyzed using Agilent human whole genome microarrays, in which 1,745 differentially expressed genes were screened. Our results indicated that EBV-induced human lymphocyte transformation was a process involving multiple genes and pathways in virus-host interactions. Hierarchical clustering analysis of the 1,745 differentially expressed genes showed that they were well distinguished between normal PBLs and LCLs, as there were significant differences between the expression patterns of the two types of host cells. There were 88 differentially expressed genes with ≥ 10 joint-edges, accounted for 81% of the total jonit-edges, which suggested that these genes may play an important role in the process of EBV transformation of B lymphocytes.

### Cell cycle and mitosis promoted differentially expressed genes

Viral genes can induce changes in the cell cycle of the host cell during the immortalization of B lymphocytes by EBV [8]. EBV latent proteins can up-regulate cyclin D1 expression in host cell, initiate the cell cycle and promote the G1-S phase transition through several signaling pathways [9,10]. EBV can also induce the up-regulation of CDK2 and CDK1 in the host cell [11]. CDK2 not only acted on the boundary of G1/S phase, but also had a key regulatory effect on S phase progression and DNA synthesis. CDK1 could promote the end of S phase and start G2 phase. The cyclin B1/CDK1 complex triggered G2/M phase transition and promoted mitosis to enter into anaphase. The activation of CDK2 was dependent on the regulation of transcription factor E2F. Research has shown that EBNA-6 and EBV transcription activation factor Zta and Rta could induce up-regulation of E2F1 in the host cell [12,13]. Annotation of differentially expressed genes and signal pathway analyses showed that up-regulated genes (CDC6, RPA3, etc.) participated in the initiation of DNA replication and DNA recombination. Therefore, we presumed that EBV might promote the progression of the cell cycle, induce B lymphocyte transformation and obtain infinite proliferation ability through up-regulating of certain cell cycle related genes.

Lacoste et al (2010) found that chromosome rearrangement might occur in subcultured early phase EBV-LCLs, mainly including chromosome deletion, chromosome fragments, dicentric chromosomes and unbalanced chromosome translocation, and aneuploidy variation may occur during the 12^th^ week post-infection [14], suggesting that EBV can induce unstable variation of the host cell genome. Gruhne et al (2009) confirmed that EBNA-1 induced DNA damage, LMP-1 inhibited DNA repair and apoptosis regulation, and EBNA-3 C down-regulated BubR1 transcription resulting host cell released CDC20 and then passived spindle checkpiont [15]. Pan et al (2009) showed that the cell spindle checkpoint could be disturbed by EBNA2 through down-regulation of MAD2 and up-regulation of PLK1, resulting in unstable chromosome variation [16]. Up-regulation of several spindle checkpoint related genes were observed in this study, including PLK1, AURKA, AURKB, NUP37, CENPA, BUB1B, CDCA8 and others. Considering that cell aneuploidy variation was mostly induced by disruption in the spindle checkpoint [17], we presumed that EBV might up-regulate the expression of a series of spindle checkpoint related genes, thus inducing change in the stability of the genome, as well as inducing B lymphocyte cell transformation and abnormal proliferation.

### Cell apoptosis inhibited differentially expressed genes

Abnormal cell proliferation has a close relation with disordered apoptosis regulation. Ng Siok-Bian et al [18] reported that LMP1 could activate NF-κB and MYC to mediate the high expression of the BIRC5 (Survivin), an inhibitor of apoptosis protein, in extranodal nasal-type NK/T cell lymphoma cells. However, LU et al [19] proposed that EBNA1 formed a complex with Sp1 or Sp1-like protein, and the cis-element of the complex combined with the BIRC5 promoter induced the up-regulation of BIRC5. In addition, Ando et al [20] reported that there existed a sequence-specific PLK1 binding region in P53, where PLK1 inhibited the transcription activity and apoptosis regulation ability of p53 by binding to this region. The high expression of BIRC5 and PLK1 in LCLs in this study suggested that EBV can inhibit B cell apoptosis and impel cell immortalization through multiple ways.

### Host immune function hindered differentially expressed genes

Among the EBV-induced down-regulated genes, several were involved in T/B cell activation of the host, suggesting that EBV regulated changes in immune function of the host during transformation of B cells. A microarray experiment conducted by Sengupta et al [21] confirmed that EBV could limit the expression of MHC-I chain-related genes in nasopharyngeal carcinoma cells. Rovedo et al [22] showed that LMP2A can bind to the SH-2 functional domain of FYN and block normal BCR signal transduction. The binding of FYN specific sequences and CD3 is required for T cell activation [23]. Gene function annotation showed that down-regulated genes, such as CD3D, CD4, CD3G, ZAP70, LCP2, ITK, etc., were involved in immunocytes activation and immune-related signal pathways, suggesting that EBV may inhibit the expression of these genes to hinder the normal immune surveillance and clearance of the host, which may allow virus-infected cells to evade immune surveillance, and ultimately induce the transformation or even malignancy of EBV-infected cells. Our previous experiments have confirmed that human-derived B-cell lymphoma could be induced in SCID mice transplanted with EBV seropositive donor’s lymphocytes [24,25], which supported the hypothesis that EBV-induced lymphomas were related to immune suppression or deficiency.

EBV BZLF1 is the switch for EBV latent state into lytic state. The expression of the BZLF1 gene is initiated from the promoter Zp, which is normally suppressed in EBV-transformed B cell. Liang et al [26] demonstrated BZLF1 gene can be activated by TGF-β through the cooperation of Smad3/Smad4 and c-Jun/c-Fos that formed a complex. The proto-oncogene product c-Fos is a component of the transcription factor AP-1. Mao et al [27] demonstrated fos protein was rarely expressed in the primary cutaneous B-cell lymphoma (PCBCL), and the result of KEGG pathway analysis showed that fos participate in B/T cell receptor signalling pathway. In this study, we hypothesized EBV may down-regulated fos to maintain its latent infection and to evade host immune surveillance and ultimately lead to the transformation of B lymphocytes.

It is noteworthy that other studies had showed that EBV could change cytokine secretion of the host cell, thereby inhibiting normal immune function, and was also an important regulatory factor for the transformation or malignant change of the B lymphocytes induced by EBV. Ehlin-Henriksson et al [28] indicated that EBNA2 and LMP1 can down-regulate the expression of CXCR4 in B-cell lymphoma cells. Chen et al [29] confirmed that EBNA-3B inhibited the expression of CXCR4 in EBV-infected B cells, thus affecting the B-cell homing and disturbing the normal immune barrier of the host. Down-regulation of cytokine-related genes-CXCR4 and CXCL1 in LCLs were also observed in this study. Therefore, we presumed that the change in expression of cytokine-related genes induced by EBV was also a regulatory factor in hindering host immune function and transformation of B lymphocytes.

In summary, EBV-induced B lymphocyte transformation was a complicated process, which involved many genes and pathways changes between virus and host interaction. Global gene expression profile analysis showed that there existed significant differences in the gene expression patterns between LCLs and normal host lymphocytes, and EBV may induce the transformation of human B lymphocytes by promoting cell cycle and mitosis, inhibiting cell apoptosis, regulating cytokine secretion, and hindering normal immune function. Our present results filtered out 38 key regulatory genes. The function and specific effect in EBV-transformed lymphocytes of the these key regulatory genes screened from EBV-transformed lymphoblastoid cell lines (LCLs) need to be further studied. This study provided important information on the molecular mechanism of lymphocyte transformation by EBV, and laid a foundation for subsequent gene validation and functional studies.

## Abbreviations

EBV, Epstein-Barr virus; PBLs, peripheral blood lymphocytes; LCLs, lymphoblastoid cell lines; Norms, normal lymphocytes; Trans, transformed lymphoblasts.

## Competing interests

The authors declare that they have no competing interests.

## Authors' contributions

DY and TY carried out the establishment of LCLs, participated in the analysis of differentially expressed genes and drafted the manuscript. DY and HF performed the bioinformatic analysis of differentially expressed genes. ZY and CA carried out the cell culture and preparation of EBV suspension. GR and WY conceived of the study, and participated in its design and coordination. All authors read and approved the final manuscript.

## References

[B1] Carbone A Gloghini A Dotti G EBV-associated lymphoroliferative disorders: classification and treatment Oncologist 2008 13 5 577 585 10.1634/theoncologist.2008-0036 18515742

[B2] Baumforth KR Young LS Flavell KJ Constandinou C Murray PG The Epstein-Barr virus and its association with human cancers Mol Pathol 1999 52 6 307 322 10.1136/mp.52.6.307 10748864 PMC395716

[B3] Zhang Y Peng J Tang Y He J Peng J Zhao Q He R Xie X Peng X Gan R The prevalence of Epstein-Barr virus infection in different types and sites of lymphomas Jpn J Infect Dis 2010 63 2 132 135 20332578

[B4] Sugimoto M Tahara H Ide T Furuichi Y Steps involved in immortalization and tumorigenesis in human B-lymphoblastoid cell lines transformed by Epstein-Barr virus Cancer Res 2004 64 10 3361 3364 10.1158/0008-5472.CAN-04-0079 15150084

[B5] Spector DL Goldman RD Leinwand LA Cells: A Laboratory Manual 1998 1 Cold Spring Harbor Laboratory, New York 71 77

[B6] Luscombe NM Babu MM Yu H Snyder M Teichmann SA Gerstein M Genomic analysis of regulatory network dynamics reveals large topological changes Nature 2004 431 7006 308 312 10.1038/nature02782 15372033

[B7] Khalyfa A Gharib SA Kim J Dayyat E Snow AB Bhattacharjee R Kheirandish-Gozal L Goldman JL Gozal D Transcriptomic analysis identifies phosphatases as novel targets for adenotonsillar hypertrophy of pediatric obstructive sleep apnea Am J Respir Crit Care Med 2010 181 10 1114 1120 10.1164/rccm.200909-1398OC 20093640 PMC2874453

[B8] Klein G Klein E Kashuba E Interaction of Epstein-Barr virus (EBV) with human B-lymphocytes Biochem Biophys Res Commun 2010 396 1 67 73 10.1016/j.bbrc.2010.02.146 20494113

[B9] Saha A Halder S Upadhyay SK Lu J Kumar P Murakami M Cai Q Robertson ES Epstein-Barr virus nuclear antigen 3 C facilitates G1-S transition by stabilizing and enhancing the function of cyclin D1 PLoS Pathog 2011 7 2 e1001275 10.1371/journal.ppat.1001275 21347341 PMC3037348

[B10] Zhang W Zeng Z Zhou Y Xiong W Fan S Xiao L Huang D Li Z Li D Wu M Li X Shen S Wang R Cao L Tang K Li G Identification of aberrant cell cycle regulation in Epstein-Barr virus-associated nasopharyngeal carcinoma by cDNA microarray and gene set enrichment analysis Acta Biochim Biophys Sin (Shanghai) 2009 41 5 414 428 10.1093/abbs/gmp025 19430707

[B11] O'Nions J Allday MJ Deregulation of the cell cycle by the Epstein-Barr virus Adv Cancer Res 2004 92 119 186 15530559 10.1016/S0065-230X(04)92006-4

[B12] Guo Q Qian L Guo L Shi M Chen C Lv X Yu M Hu M Jiang G Guo N Transactivators Zta and Rta of Epstein-Barr virus promote G0/G1 to S transition in Raji cells: a novel relationship between lytic virus and cell cycle Mol Immuno 2010 47 9 1783 1792 10.1016/j.molimm.2010.02.017 20338640

[B13] Kashuba E Yurchenko M Yenamandra SP Snopok B Isaguliants M Szekely L Klein G EBV-encoded EBNA-6 binds and targets MRS18-2 to the nucleus, resulting in the disruption of pRb-E2F1 complexes Proc Natl Acad Sci USA 2008 105 14 5489 5494 10.1073/pnas.0801053105 18391203 PMC2291094

[B14] Lacoste S Wiechec E Dos Santos Silva AG Guffei A Williams G Lowbeer M Benedek K Henriksson M Klein G Mai S Chromosomal rearrangements after ex vivo Epstein-Barr virus (EBV) infection of human B cells Oncogene 2010 29 4) 503 515 19881539 10.1038/onc.2009.359

[B15] Gruhne B Kamranvar SA Masucci MG Sompallae R EBV and genomic instability–a new look at the role of the virus in the pathogenesis of Burkitt's lymphoma Semin Cancer Biol 2009 19 6 394 400 10.1016/j.semcancer.2009.07.005 19619655

[B16] Pan SH Tai CC Lin CS Hsu WB Chou SF Lai CC Chen JY Tien HF Lee FY Wang WB Epstein-Barr virus nuclear antigen 2 disrupts mitotic checkpoint and causes chromosomal instability Carcinogenesis 2009 30 2 366 375 19126642 10.1093/carcin/bgn291

[B17] McIntosh JR Structural and mechanical control of mitotic progression Cold Spring Harb Symp Quant Biol 1991 56 613 619 10.1101/SQB.1991.056.01.070 1819511

[B18] Ng SB Selvarajan V Huang G Zhou J Feldman AL Law M Kwong YL Shimizu N Kagami Y Aozasa K Salto-Tellez M Chng WJ Activated oncogenic pathways and therapeutic targets in extranodal nasal-type NK/T cell lymphoma revealed by gene expression profiling J Pathol 2011 223 4 496 510 10.1002/path.2823 21294123

[B19] Lu J Murakami M Verma SC Cai Q Haldar S Kaul R Wasik MA Middeldorp J Robertson ES Epstein-Barr Virus nuclear antigen 1 (EBNA1) confers resistance to apoptosis in EBV-positive B-lymphoma cells through up-regulation of survivin Virology 2011 410 1 64 75 10.1016/j.virol.2010.10.029 21093004 PMC4287362

[B20] Ando K Ozaki T Yamamoto H Furuya K Hosoda M Hayashi S Fukuzawa M Nakagawara A Polo-like Kinase 1 (Plk1) Inhibits p53 Function by Physical Interaction and Phosphorylation J Biol Chem 2004 279 24 25549 25561 10.1074/jbc.M314182200 15024021

[B21] Sengupta S den Boon JA Chen IH Newton MA Dahl DB Chen M Cheng YJ Westra WH Chen CJ Hildesheim A Sugden B Ahlquist P Genome-wide expression profiling reveals EBV-associated inhibition of MHC class I expression in nasopharyngeal carcinoma Cancer Res 2006 66 16 7999 8006 10.1158/0008-5472.CAN-05-4399 16912175

[B22] Rovedo M Longnecker R Epstein-Barr Virus Latent Membrane Protein 2A Preferentially Signals through the Src Family Kinase Lyn J Virol 2008 82 17 8520 8528 10.1128/JVI.00843-08 18579586 PMC2519656

[B23] Filby A Seddon B Kleczkowska J Salmond R Tomlinson P Smida M Lindquist JA Schraven B Zamoyska R Fyn regulates the duration of TCR engagement needed for commitment to effector function J Immunol 2007 179 7 4635 4644 17878361 10.4049/jimmunol.179.7.4635

[B24] Gan R Yin Z Liu T Wang L Tang Y Song Y Cyclosporine A effectively inhibits graft-versus-host disease during development of Epstein-Barr virus-infected human B cell lymphoma in SCID mouse Cancer Sci 2003 94 9 796 801 10.1111/j.1349-7006.2003.tb01521.x 12967478 PMC11160143

[B25] Gan R Xie X He J Liu X Hong L Tang Y Liu F Xie H Gene analysis of Epstein-Barr virus-associated lymphomas in hu-PBL/SCID chimeras Tumori 2010 96 3 465 472 20845810 10.1177/030089161009600315

[B26] Liang CL Chen JL Hsu YP Ou JT Chang YS Epstein-Barr virus BZLF1 gene is activated by transforming growth factor-beta through cooperativity of Smads and c-Jun/c-Fos proteins J Biol Chem 2002 277 26 23345 23357 10.1074/jbc.M107420200 11971895

[B27] Mao X Orchard G Abnormal AP-1 protein expression in primary cutaneous B-cell lymphomas Br J Dermatol 2008 159 1 145 151 10.1111/j.1365-2133.2008.08579.x 18460027

[B28] Ehlin-Henriksson B Liang W Cagigi A Mowafi F Klein G Nilsson A Changes in chemokines and chemokine receptor expression on tonsillar B cells upon Epstein-Barr virus infection Immunology 2009 127 4 549 557 10.1111/j.1365-2567.2008.03029.x 19604305 PMC2729532

[B29] Chen A Zhao B Kieff E Aster JC Wang F EBNA-3B- and EBNA-3 C-regulated cellular genes in Epstein-Barr virus-immortalized lymphoblastoid cell lines J Virol 2006 80 20 10139 10150 10.1128/JVI.00854-06 17005691 PMC1617319

